# Clinical efficacy and safety of transvaginal natural orifice transluminal endoscopic surgery for benign adnexal disease: a prospective trial

**DOI:** 10.1186/s12905-024-03226-5

**Published:** 2024-07-05

**Authors:** Jinbowen Yan, Dan Zhou, Shuo Zhang, Bo Zhang, Xunyuan Tuo, Qingwei Meng, Qiubo Lv

**Affiliations:** 1 Department of obstetrics and Gynecology, Beijing Hospital, National Center of Gerontology, Institute of Geriatric Medicine, Chinese Academy of Medical Sciences, Beijing, P.R. China; 2 Gansu Provincial Maternal and Child Health Center Gansu Provincial Central Hospital, Lanzhou, Gansu P.R. China

**Keywords:** vNOTES, Ovarian cyst, Prospective randomized controlled trial

## Abstract

**Background:**

There is a scarcity of prospective clinical research evidence regarding the utilization of transvaginal natural orifice translumenal endoscopic surgery (vNOTES) as a treatment option for ovarian cysts. The objective of this study was to assess the feasibility and safety of employing vNOTES for the management of ovarian cysts.

**Methods:**

Our study included women between the ages of 18 and 70 who intended to undergo surgical intervention for benign lesions. Stratified blocked randomization was employed to allocate participants into groups. The main objective was to assess whether the assigned group adhered to the recommended surgical technique for ovarian cystectomy or adnexectomy, without any deviation to alternative surgical methods.

**Results:**

A total of 196 patients were included in the study, with all surgeries in each group being conducted according to the assigned procedures. Among them, the ovarian cystectomy layer included 58 cases in the vNOTES group and 58 cases in the conventional laparoscopy (CL) groups. The adnexectomy layer included 40 cases in the vNOTES group and 40 cases in the CL group. Utilizing a sensitivity analysis, the two-sided 95% lower confidence limit was determined to be 5.5% for the disparity in proportions between the vNOTES groups and CL groups. These lower limits fell below the predetermined non-inferiority margin of 10%.

**Conclusions:**

The study findings demonstrate that vNOTES was not inferior to CL in terms of adnexectomy or ovarian cystectomy. vNOTES can be considered a more minimally invasive surgical approach, as it results in reduced postoperative pain, faster recovery, and absence of visible incisions. Overall, vNOTES proves to be a safe, feasible, and less invasive treatment option.

**Trial registration:**

This study retrospectively registered with the China Clinical Trial Registry with the registration number ChiCTR2100052223(22-10-2021).

## Backgroud

Ovarian cysts are frequently occurring nonmalignant neoplasms within the female reproductive system. Due to a significant number of patients being asymptomatic and undiagnosed, the exact prevalence of ovarian cysts remains uncertain [1]. These cysts have the potential to result in urgent gynecological complications, including hemorrhage, rupture, and torsion [2]. Surgical intervention is the preferred approach for managing persistent or enlarging ovarian cysts [3]. Laparoscopic surgery is generally regarded as superior to laparotomy for the treatment of benign ovarian masses [4]. Conventional laparoscopy (CL) utilizing a transabdominal multiport approach is a well-established technique employed in the management of benign adnexal diseases. In the context of CL, efforts have been undertaken to minimize the quantity of puncture holes, as each puncture hole amplifies the likelihood of encountering complications such as hemorrhaging, infection, nerve impairment, and hernia development.

Natural orifice translumenal endoscopic surgery (NOTES) represents a significant progression in the field of minimally invasive surgery, wherein the thoracic, abdominal, and pelvic cavities are accessed through natural orifices such as the oral cavity, vagina, anus, and others, enabling the performance of corresponding surgical procedures. In 2004, Kalloo et al. successfully employed a porcine model to establish endoscopic entry into the peritoneal cavity via the natural orifice of the stomach, thus validating the viability of NOTES through subsequent animal experiments [5–9]. The utilization of transgastric, transvaginal, and transrectal approaches has become prevalent in surgical procedures, including cholecystectomy [10, 11]. Among these approaches, transvaginal is frequently employed in diverse gynecological conditions due to the anatomical benefits offered by the vagina [12].

However, the field of gynecology lacks sufficient prospective clinical research evidence regarding the use of transvaginal natural orifice translumenal endoscopic surgery (vNOTES) for the treatment of ovarian cysts. Consequently, this study endeavors to investigate the safety and feasibility of vNOTES in treating ovarian cysts through a prospective randomized controlled study (RCT).

## Methods

### Study design and participants

A randomized controlled trial (RCT) was conducted at Beijing Hospital and registered with the China Clinical Trial Registry under the registration number ChiCTR2100052223. Patient enrollment took place from August 1, 2019, to February 1, 2022. The study received approval from the ethics committee of Beijing Hospital (no.2019BJYYEC-250-02). The entire study was funded by the Beijing Science and Technology Commission.

This study considered women aged 18 to 70 years with benign ovarian cysts who were scheduled to undergo ovarian cystectomy or adnexectomy as eligible participants. The inclusion criteria consisted of the following: ① being between 18 and 70 years old, ② having a preoperative risk of malignancy index (RMI) indicating a high likelihood of a benign condition, ③ having regular postoperative follow-up appointments to obtain information at 1 month, 3 months, and 6 months after the operation, and ④ providing informed consent by agreeing to participate in the study and signing a consent form. The exclusion criteria encompassed the following: ① Women identifying as asexual, ② Individuals with vaginal stenosis, ③ Participants with vaginal infections caused by bacteria, fungi, trichomoniasis, mycoplasma, chlamydia, or other pathogens, ④ Individuals with a history of rectal surgery,⑤ Women with ovarian cysts larger than 10 cm, ⑥ Suspected cases of rectovaginal endometriosis, ⑦ Suspected cases of malignancy,⑧ Pregnant individuals, and ⑨ Those unable to partake in postoperative follow-up.

This study employed a stratified block randomization technique, whereby all patients were categorized through random assignment within each stratum. Patients were then divided into two groups, namely the adnexectomy group and the ovarian cystectomy group, based on the extent of surgical resection. A random number table was generated by statistical experts using specialized software, and subsequently utilized to create opaque envelopes for random grouping. The enrolled subjects were allocated to either the experience group for vNOTES or the control group for CL in accordance with their order of enrollment. The process of randomization was conducted individually for participants within each stratum.

### Procedures

The operations on all subjects included in the study were conducted by the same group of experienced doctors with clinical expertise at the research center, irrespective of their assignment to the ovarian cystectomy or adnexectomy groups. The surgical technique employed for CL involved the conventional approach of inserting the trocar through the umbilical incision and introducing the laparoscope. During the procedure, a 1 cm incision was made 3 cm medial to the left and right anterior superior iliac spines, and trocars of 5 mm and 10 mm were inserted into the abdominal cavity. Following the operation, the incision was closed using either sutures or adhesive. The surgical technique known as vNOTES entails making a 2–3 cm incision on the mucosa of the posterior vaginal fornix. Following verification of the absence of bowel injury, a single-port laparoscopic port was introduced, allowing for the insertion of a laparoscope and operating instruments to carry out the procedure. The intravaginal surgical procedures closely resembled those of standard laparoscopy. The excised specimens were securely contained within custom-made specimen bags and were entirely extracted through the enlarged incision in the posterior vaginal fornix. The closure of the posterior fornix of the vagina was performed under direct visualization following the surgical procedure.

### Outcomes measurements

The main objective of the study was to assess the occurrence of adnexectomy or ovarian cystectomy using the designated surgical approach. Additional measures of interest encompassed the length of the surgical procedure (from initiation to completion of incision suturing), intraoperative blood loss (quantified by the volume of blood collected via suction during the operation), and pain levels reported on the visual analogue scale (VAS) within the first 24 h and week post-surgery [13]. Short Form 36-item Health Survey (SF-36) [14] and Female Sexual Function Index (FSFI) [15] were used to evaluate the quality of life and sexual function of patients before surgery, 3 months post-surgery, and 6 months post-surgery.

The safety measures were evaluated by examining the occurrences of intraoperative and postoperative adverse events. Intraoperative adverse events encompassed shock, damage to major blood vessels (with blood volume exceeding 1000 mL), rectal injury, and ureteral injury. Postoperative adverse events included infection rate, rate of postoperative complications (such as wound dehiscence, suppuration, and rectovaginal fistula), readmission rate within 6 weeks, and dyspareunia at 1, 3, and 6 months following the surgery.

### Sample size calculation

Based on our previous surgical experience, the success rate of transvaginal single-port laparoscopic adnexal surgery and conventional transabdominal incision laparoscopic surgery was approximately 94%, as determined through the utilization of a non-inferiority test. The non-inferiority margin for this study was established at 10%, with an equal ratio of 1:1 between the two groups. The study employed a one-sided test with a significance level of 0.025 and a statistical power of 84%. The effective sample size of 89 pairs was estimated using the PASS2008 software. Considering a dropout rate of 10%, the final calculated sample size was determined to be 98 pairs.

### Statistical analysis

All analyses were based on the intention-to-treat principle. The measurement data has been represented as mean and standard deviation, and the count data has been represented as the percentage of cases. A group t-test or nonparametric test was used to compare measurement data between groups; a chi-square test or exact probability test was used for enumeration data, and a two-sided test was used. Data was analyzed using SPSS 26, and *P* < 0.05 was considered statistically significant. The 95% confidence interval (CI) of the surgical success rate and the difference between the two groups was calculated. The lower limit of the confidence interval and the 10% non-inferiority margin were compared to determine whether non-inferiority could be achieved.

## Results

**Fig. 1:**
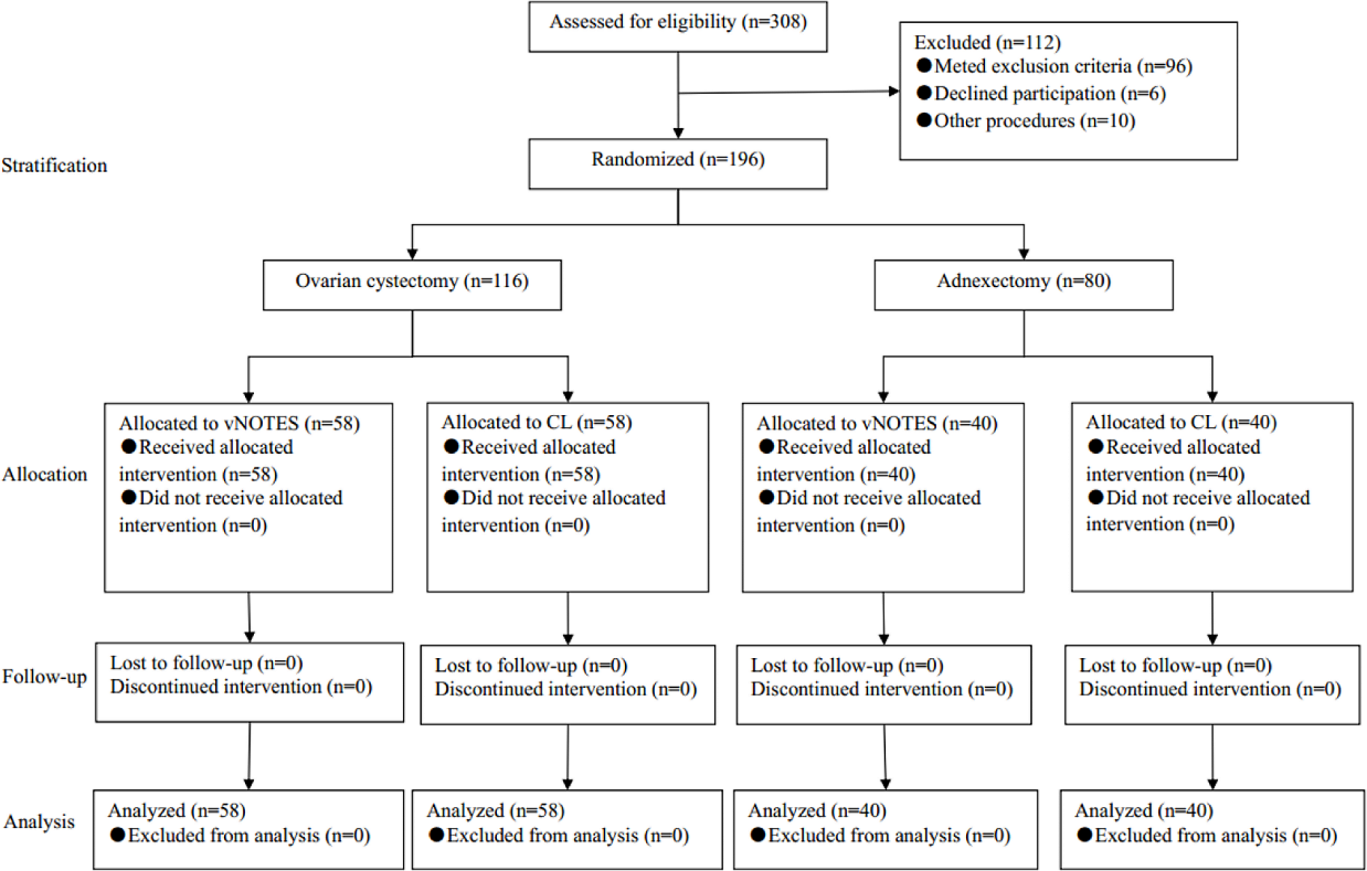
CONSORT flow chart

Figure 1 depicts the CONSORT flow chart illustrating the trial. Within the period of August 2019 to February 2022, a total of 196 patients who fulfilled the inclusion criteria actively participated in this study and provided informed consent. Among these patients, 116 individuals underwent ovarian cystectomy while 80 individuals underwent adnexectomy. Random assignment was conducted, with the vNOTES group assigned to the ovarian cystectomy patients and the CL group assigned to the adnexectomy patients. All patients had data available for the primary outcome. Baseline characteristics for both the ovarian cystectomy and adnexectomy groups were comparable between the vNOTES and CL groups, as indicated in Tables 1 and 2.

**Table 1:** Baseline characteristics (adnexectomy groups)*

	vNOTES *n* = 40	CL *n* = 40
Age(years)**	58.8 ±6.2	56.8 ±6.7
**BMI (kg/m** ^**2**^ )**	24.3 ±3.1	24.1 ±3.0
Lesion (cm)**	4.7 ±1.1	5.2 ±1.5
**Prior pelvic disease, n(%)**	8(20%)	6(15%)
**No. of vaginal births, n(%)**	33(82%)	29(72%)
**Pathological type**		
Serous cystadenoma	23(57.5%)	21(52.5%)
Fibroma	9(22.5%)	11(27.5%)
Mesosalpinx cyst	3(7.5%)	4(10%)
Theca cell tumor	5(12.5%)	4(10%)
SF-36**	120.1 ±11.2	117.2 ±14.3
FSFI**	9.2 ±10.8	15.4 ±21.8

CL: conventional laparoscopy; SF: Short Form 36-item Health Survey; FSFI: Female Sexual Function Index

*There were no significant differences (*P* < 0.05) between the two groups

**Mean and standard deviation

**Table 2:** Baseline characteristics (ovarian cystectomy groups)*

	vNOTES *n* = 58	CL *n* = 58
Age(years)**	33.8 ±6.9	32.1 ±6.7
**BMI (kg/m** ^**2**^ )**	20.9 ±2.8	21.3 ±3.6
Lesion (cm)**	6.6 ±1.6	6.0 ±2.0
**Prior pelvic disease, n (%)**	11(18%)	12(20%)
**No. of vaginal births, n (%)**	20(34%)	15(25%)
**Pathological type**		
Teratoma	33(57%)	30(52%)
Serous cystadenoma	18(31%)	17(29%)
Mesosalpinx cyst	5(9%)	7(12%)
endometriotic cyst	2(3%)	4(6%)
SF-36**	123.6 ±10.8	121.6 ±11.0
FSFI**	19.2 ±11.0	17.1 ±11.3

CL: conventional laparoscopy; SF: Short Form 36-item Health Survey; FSFI: Female Sexual Function Index

*There were no significant differences (*P* < 0.05) between the two groups

**Mean and standard deviation

### Primary outcome

The surgeries were conducted using the designated technique for all patients within each group. Due to the absence of surgical transfers, it was not possible to assess the disparities between the two surgical modalities. Consequently, a sensitivity analysis was conducted to evaluate the success rate of the surgical procedures. In one instance, a patient in the experimental groups underwent a conversion to alternative surgical methods during the operation, while all surgeries in the control groups were executed successfully. Following the analysis, the estimation of the two-sided 95% lower confidence limit for the disparity in proportions in both the experimental and control groups was determined to be 5.5% (95%CI -5.5 to 2.8%). It is noteworthy that the upper limits fell below the predetermined 10% non-inferiority threshold.

### Secondary outcomes

**Fig. 2:**
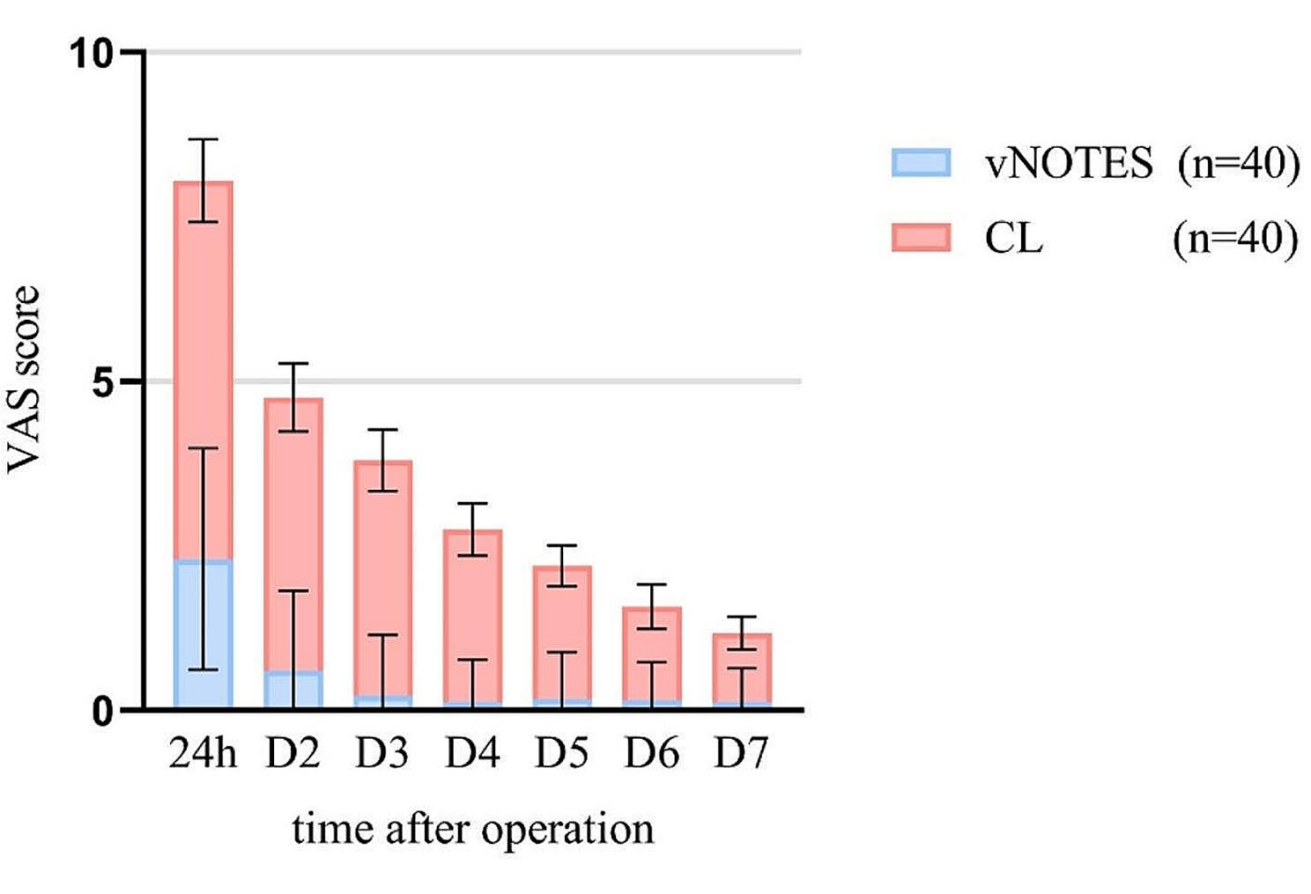
Pain assessment of adnexectomy groups

The results of the primary secondary outcomes of this trial are displayed in Tables 3 and 4. In the adnexectomy groups, the average duration of vNOTES was found to be shorter compared to CL, with a mean difference of -0.8 min (95%CI -11.9 to 10.2 min; *P* > 0.05). Additionally, the mean intraoperative blood loss of vNOTES was observed to be lower than CL, with a mean difference of -5.0 ml (95%CI -14.8 to -4.8 ml; *P* > 0.05). However, neither of these differences were found to be statistically significant. The duration of postoperative exhaust time for vNOTES was found to be significantly shorter compared to the CL group, with a mean difference of -20.4 h (95%CI -23.0 to -17.8 h; *P* < 0.05). Figure 2 illustrates the changing trend and significant differences in postoperative pain scores between the two groups at various time points. The vNOTES group exhibited significantly higher scores (*P* < 0.05) on the SF-36 scale at 3 and 6 months after the operation compared to the CL group. However, no statistically significant difference was observed between the two groups on the FSFI scale during the 3-month and 6-month follow-ups. These results are presented in Table 3. Notably, no complications were reported in the vNOTES group, either during or after the surgery. Conversely, 14 patients in the CL group experienced postoperative abdominal distension and shoulder pain.

**Fig. 3:**
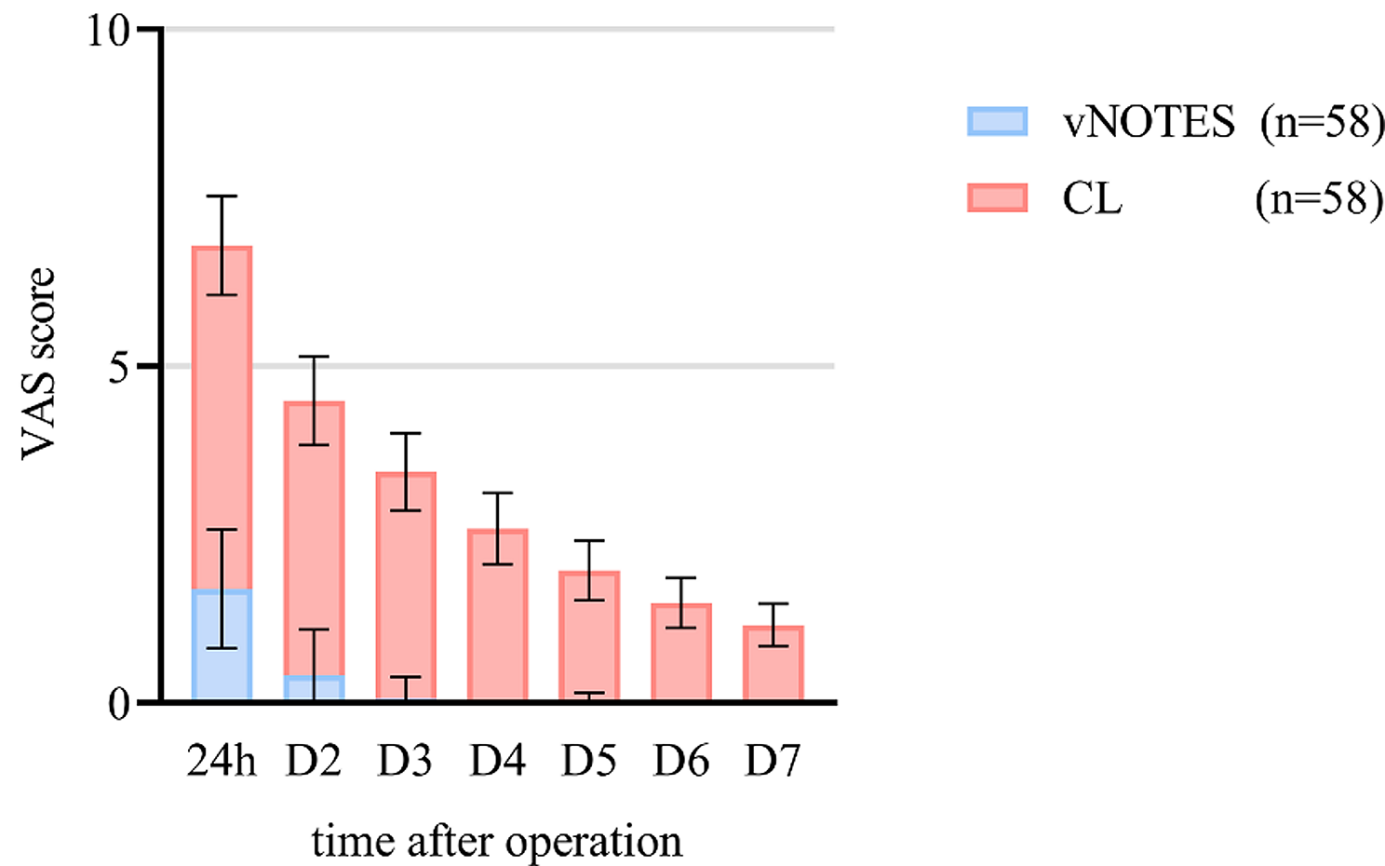
Pain assessment of ovarian cystectomy groups

In the ovarian cystectomy groups, the average duration of vNOTES was found to be significantly shorter compared to CL, with a mean difference of -11.2 min (95%CI -18.7 to -3.7 min; *P* < 0.05). The mean intraoperative blood loss of vNOTES was lower than CL, but this difference did not reach statistical significance, with a mean difference of -6.9 ml (95%CI -14.0 to -0.07 ml; *P* > 0.05). However, the postoperative exhaust time of vNOTES was significantly shorter than that of CL, with a mean difference of -18.7 h (95%CI -22.8 to -14.7 h; *P* < 0.05). Figure 3 illustrates the dynamic pattern and disparity in postoperative pain scores among the two groups, with statistically significant differences observed at each node (*P* < 0.05). Notably, the vNOTES group exhibited significantly higher scores (*P* < 0.05) on both the SF-36 and FSFI scales at 3 and 6 months post-operation compared to the CL group. These noteworthy results are further detailed in Table 4. Importantly, no complications were encountered in the vNOTES group throughout the entire surgical process. Within the CL group, two patients exhibited symptoms of a low-grade fever, while fourteen patients experienced abdominal distension and shoulder pain. Additionally, two patients presented with suppuration stemming from the umbilical puncture.

**Table 3:** Main outcomes (adnexectomy groups)

	vNOTES *n* = 40	CL *n* = 40	*P*
**Duration of surgery (minutes), mean ± SD**	69.4 ±27.2	70.2 ±22.5	0.41
**Intraoperative blood loss(ml), mean ± SD**	26.6 ±15.8	31.6 ±26.6	0.31
**Anal exhaust time(hours), mean ± SD**	12.6 ±4.0	33.0 ±7.2	< 0.05
**Complications**			
Intra-operative, n (%)	0	0	
Postoperative, n (%)	0	14(35.0%)	
**Postoperative SF**			
3 month	125.1 ±9.6	117.8 ±11.2	< 0.05
6 month	123.6 ±9.9	118.9 ±11.3	< 0.05
**Postoperative FSFI**			
3 month	8.4 ±9.9	9.6 ±19.9	0.72
6 month	8.6 ±9.6	10.2 ±19.7	0.63

**Table 4:** Main outcomes (ovarian cystectomy groups)

	vNOTES *n* = 58	CL *n* = 58	*P*
**Duration of surgery (minutes), mean ± SD**	64.2 ±21.4	75.5 ±19.1	< 0.05
**Intraoperative blood loss(ml), mean ± SD**	27.2 ±17.7	34.2 ±20.4	0.052
**Anal exhaust time(hours), mean ± SD**	19.2 ±8.1	38.0 ±13.4	< 0.05
**Complication**			
Intra-operative, n (%)	0	0	
Postoperative, n (%)	0	19(32.7%)	
**Postoperative SF**			
3 month	128.6 ±8.7	118.7 ±9.2	< 0.05
6 month	127.5 ±9.8	119.3 ±10.0	< 0.05
**Postoperative FSFI**			
3 month	22.1 ±9.0	10.1 ±10.5	< 0.05
6 month	25.7 ±9.4	9.4 ±10.5	< 0.05

## Discussion

In this prospective clinical study, the authors have successfully demonstrated the feasibility and safety of Vnotes in the management of gynecological adnexal diseases, as evidenced by follow-up observations. A total of 196 patients were enrolled in the study, all of whom underwent the designated procedures. The obtained results align with the findings reported by Baekelandt J et al., who introduced a novel approach for conducting ovarian cystectomy [16]. The pivotal aspect contributing to the efficacy of vNOTES lies in the precise opening of the posterior fornix of the vagina to gain access to the abdominal cavity, with two crucial factors influencing the execution of this technique. Prior to surgery, it is imperative to conduct a thorough assessment of the posterior vaginal fornix through bimanual and trimanual examination, as well as transvaginal ultrasound. This examination should encompass evaluating the presence of sufficient vaginal access, the existence of tender nodes in the posterior vaginal fornix and posterior uterine wall, and the potential adhesion or closure of the uterorectal socket. Additionally, it is crucial to make an informed decision regarding the appropriate positioning of the incision in the posterior vaginal fornix. The proximity of the side adjacent to the cervix may result in an indistinct anatomical structure and hinder access to the abdominal cavity, whereas the side in close proximity to the rectum poses a risk of damaging the intestinal tract [17]. Consequently, certain scholars have introduced the notion of performing a “safety triangle” procedure as a potential solution.

Fortunately, no instances of rectal injury were observed among the patients in the vNOTES group during our study. Within this group, three participants with uncomplicated chocolate cysts successfully underwent the procedure. It is noteworthy that the recto-uterine pit, being the lowest point within the pelvic cavity, can serve as a site for the accumulation of ruptured bleeding and fluid originating from benign ovarian tumors. Additionally, a history of pelvic and abdominal surgeries may result in the development of severe pelvic adhesions [18]. These factors can potentially impede the entry of the posterior fornix into the abdominal cavity. Hence, it is imperative to conduct a thorough and extensive assessment of the patient’s posterior fornix prior to surgical intervention. Closure of the uterorectal fossa may occur as a result of prior pelvic surgery, documented pelvic adhesions, endometriosis lesions, pelvic inflammatory disease, or any other etiology. Consequently, individuals with a medical history of pelvic-abdominal surgery and posterior fornix adhesions were deliberately omitted from the research study. This exclusionary criterion may contribute to the observed 100% surgical success rate within the vNOTES group.

No statistically significant differences were observed in the duration of surgery and intraoperative blood loss between the vNOTES and CL groups in the adnexectomy cohorts. However, in the ovarian cystectomy cohorts, the mean duration of vNOTES was significantly shorter compared to CL. These findings align with the results reported by Lee CL et al. [19], but differ from those reported by Kaya C et al. [20]. Based on our study’s clinical practice experience and expertise in vaginal surgery, we observed a significant reduction in operation time after independently performing approximately 40 vNOTES cases. In their study on the learning curve of vNOTES for adnexal surgery, Huang YT et al. demonstrated that a gynecologist with appropriate training could achieve proficiency in surgical procedures after completing 36 vNOTES cases [21].

In comparison to the CL group, patients in the vNOTES group exhibited prompt anal exhaust following surgery, aligning with the observations made by Zhang J et al. [22]. This correlation may be attributed to the fact that the vNOTES procedure is performed in the lower pelvic region, allowing for operation under low intra-abdominal pressure or without pneumoperitoneum. Pneumoperitoneum exerts minimal impact on intestinal function, enabling certain patients to engage in bedside activities merely 6 h post-operation, thereby facilitating the restoration of intestinal function. In order to establish a secure surgical operating space and prevent harm to surrounding organs and tissues, it is necessary for CL to maintain an optimal level of CO_2_ to create an ideal pneumoperitoneum environment. It has been theorized that postoperative pain following laparoscopic surgery may be attributed to the retention of CO_2_ in the abdominal cavity, which subsequently irritates the phrenic nerve and diaphragm, resulting in referred pain in the shoulder and upper abdomen [23]. Despite appropriate postoperative deflation, our study observed that 28 patients experienced postoperative shoulder pain in the CL group. No reports of shoulder pain potentially associated with the vaginal incision and sufficient deflation were found in the vNOTES. Subsequent assessments indicated that diaphragm and shoulder pain could impede patients’ regular breathing patterns, but this issue gradually resolved within one month after the surgery.

The utilization of NOTES demonstrated considerable benefits in terms of postoperative pain management and wound recovery. In contrast to the mucosal nature of the posterior fornix incision, the CL involves a skin incision that often leads to noticeable scarring, particularly in the umbilicus region. The deep wound in the umbilicus hinders the patient’s ability to effectively care for the wound after being discharged from the hospital. Consequently, this situation leads to the accumulation of secretions and bacterial colonization, creating a microenvironment that promotes bacterial reproduction while impeding wound healing. In the present study, it was observed that two patients belonging to the CL group experienced infection and purulence of the umbilical port wound following the surgical procedure.

Both CL and vNOTES are considered minimally invasive surgical techniques, and there were no significant alterations in the overall health status of patients following surgery when compared to their preoperative condition. The health assessment of patients in our study was conducted using the Health Survey Short Form SF-36. The findings revealed that the vNOTES group exhibited superior postoperative health outcomes at both the 1-month and 6-month intervals compared to the CL group. This disparity may be attributed to reduced surgical trauma, diminished pain, and expedited postoperative recovery associated with the vNOTES approach. In the groups undergoing ovarian cystectomy, the vNOTES group exhibited superior postoperative sexual function outcomes compared to the control group, with a statistically significant difference. These findings align with the research conducted by Linke GR et al. [24], which similarly reported no evidence of infertility or dyspareunia as long-term complications following vNOTES [25]. However, in the context of absorbable sutures and wound healing, it is recommended that patients abstain from engaging in sexual intercourse for a minimum duration of six weeks following surgery.

It is important to acknowledge several limitations inherent in our study. Firstly, this trial was conducted at a single center, with all procedures performed by a single expert surgeon, thereby restricting the generalizability of our findings. Additionally, blinding was not feasible in surgical trials due to the unique nature of the procedure. Furthermore, the postoperative follow-up results may be subject to bias stemming from patients’ limited understanding of the surgical methodology. Furthermore, the scope of this study is restricted to the Chinese population, thereby potentially compromising the generalizability of the findings on a global scale.

## Conclusion

vNOTES has been found to be comparable to CL in terms of therapeutic efficacy and safety for treating benign ovarian tumors. The non-inferiority of vNOTES to CL has been demonstrated in performing adnexectomy or ovarian cystectomy without the need for conversion. Furthermore, vNOTES surgery exhibits reduced invasiveness, fewer reports of pain, and quicker recovery time when compared to CL. It is crucial to emphasize the importance of conducting a thorough evaluation of pelvic adhesions prior to surgery. The utilization of the vNOTES approach is contraindicated for patients with posterior fornix closure. However, the vNOTES approach exhibits simplicity and ease of adoption for surgeons who possess prior proficiency in both laparoscopic surgery and vaginal surgery.

## Data Availability

The datasets used during the current study are available from the corresponding author upon reasonable request.

## Abbreviations

CL: Conventional Laparoscopy
NOTES: Natural Orifice Translumenal Endoscopic Surgery
vNOTES: Transvaginal Natural Orifice Translumenal Endoscopic Surgery
RCT: Randomized Controlled Study
RMI: Risk of Malignancy Index
VAS: Visual Analogue Scale
SF-36: Short Form 36-item Health Survey
FSFI: Female Sexual Function Index
CI: Confidence Interval
